# Development and Validation of a Deep Learning Model for Detection of Allergic Reactions Using Safety Event Reports Across Hospitals

**DOI:** 10.1001/jamanetworkopen.2020.22836

**Published:** 2020-11-16

**Authors:** Jie Yang, Liqin Wang, Neelam A. Phadke, Paige G. Wickner, Christian M. Mancini, Kimberly G. Blumenthal, Li Zhou

**Affiliations:** 1 Division of General Internal Medicine and Primary Care, Brigham and Women’s Hospital, Boston, Massachusetts; 2 Harvard Medical School, Boston, Massachusetts; 3 Division of Rheumatology, Allergy, and Immunology, Massachusetts General Hospital, Boston; 4 Division of Allergy and Clinical Immunology, Brigham and Women’s Hospital, Boston, Massachusetts

## Abstract

**Importance:**

Although critical to patient safety, health care–related allergic reactions are challenging to identify and monitor.

**Objective:**

To develop a deep learning model to identify allergic reactions in the free-text narrative of hospital safety reports and evaluate its generalizability, efficiency, productivity, and interpretability.

**Design, Setting, and Participants:**

This cross-sectional study analyzed hospital safety reports filed between May 2004 and January 2019 at Brigham and Women’s Hospital and between April 2006 and June 2018 at Massachusetts General Hospital in Boston. Training and validating a deep learning model involved extracting safety reports using 101 expert-curated keywords from Massachusetts General Hospital (data set I). The model was then evaluated on 3 data sets: reports without keywords (data set II), reports from a different time frame (data set III), and reports from a different hospital (Brigham and Women’s Hospital; data set IV). Statistical analyses were performed between March 1, 2019, and July 18, 2020.

**Main Outcomes and Measures:**

The area under the receiver operating characteristic curve and area under the precision-recall curve were used on data set I. The precision at top-k was used on data sets II to IV.

**Results:**

A total of 299 028 safety reports with 172 854 patients were included. Of these patients, 86 544 were women (50.1%) and the median (interquartile range [IQR]) age was 59.7 (43.8-71.6) years. The deep learning model achieved an area under the receiver operating characteristic curve of 0.979 (95% CI, 0.973-0.985) and an area under the precision-recall curve of 0.809 (95% CI, 0.773-0.845). The model achieved precisions at the top 100 model-identified cases of 0.930 in data set II, 0.960 in data set III, and 0.990 in data set IV. Compared with the keyword-search approach, the deep learning model reduced the number of cases for manual review by 63.8% and identified 24.2% more cases of confirmed allergic reactions. The model highlighted important words (eg, *rash*, *hives,* and *Benadryl*) in prediction and extended the list of expert-curated keywords through an attention layer.

**Conclusions and Relevance:**

This study showed that a deep learning model can accurately and efficiently identify allergic reactions using free-text narratives written by a variety of health care professionals. This model could be used to improve allergy care, potentially enabling real-time event surveillance and guidance for medical errors and system improvement.

## Key Points

**Question:**

Can a deep learning model applied to the free-text narrative of hospital safety event reports identify allergic reactions?

**Findings:**

In this cross-sectional study of 299 028 hospital safety reports involving 172 854 patients, a deep learning model was developed and validated on the basis of a subset of the reports and reached an area under the receiver operating characteristic curve of 0.979 for identifying allergic reactions. Compared with the keyword-search approach, the model identified 24.2% more cases of confirmed allergic reactions and reduced the need for manual review by 63.8%.

**Meaning:**

Results of this study suggest that deep learning can improve the accuracy and efficiency of the allergic reaction identification process, which may facilitate future real-time patient safety surveillance and guidance for medical errors and system improvement.

## Teaser

This cross-sectional study describes the development and evaluation of a deep learning model to identify allergic reaction in the free-text narrative of hospital safety reports.

## Introduction

Allergic reactions to medications, foods, and other health care products are becoming increasingly common in the United States, with up to 36% of patients reporting drug allergies and 4% to 10% reporting food allergies.^1,2,3^ At least 1 in 5 of these reported allergies are allergic reactions (ie, hypersensitivities) with symptoms ranging from minor rashes to severe anaphylaxis.^4^ Patients in health care settings are at particularly high risk for developing an allergic reaction given their many new exposures.^5,6,7^ Given that allergic reactions can cause patient harm^8^ and result in malpractice litigation,^9,10,11^ timely allergic event detection, monitoring, and characterization are critical for improving health care quality and patient safety.^6,12^

Hospital safety event reporting systems, which collect voluntarily reported safety event data from frontline personnel, are integral to the detection of patient safety signals in health care.^13,14^ Safety reports contain a large amount of data, but still lacking are processes to analyze them in a manner that allows for timely feedback to health care professionals or actions to prevent similar future events.^15,16,17^ The rarity of allergic reactions makes it unlikely to be classified as a separate safety event category that is easily detected and monitored. Manual review of keyword-filtered safety reports is time- and labor-intensive; overly sensitive parameters are associated with false-positive cases,^12^ and an overly restricted keyword repertoire is associated with missed cases.^18,19^ In a previous study, 101 keywords related to allergic symptoms, treatments, and culprits were used to search 128 753 hospital safety reports over a 10-year period.^12^ Among the 9107 reports retrieved by keywords, only 431 reports (4.7%) were confirmed as true allergic reactions, and it was unclear how many cases were missed.^12^ Machine learning studies have detected adverse drug reactions (ADRs) from electronic health records,^5,20,21^ safety reports,^22^ and social media data,^23^ but few studies have focused on allergic reaction identification.

In this study, we developed an artificial intelligence method, a hierarchical attention-based deep neural network (DNN), that automatically reads the free-text description of voluntarily filed hospital safety reports and identifies cases describing allergic reactions. We assessed the model’s performance using a manually labeled data set and evaluated the generalizability, efficiency, productivity, and interpretability of the model using new data without keywords as well as data from a different time frame and hospital.

## Methods

This cross-sectional study was approved by the Mass General Brigham Institutional Review Board, which waived the informed consent requirement from study participants because of secondary use of hospital safety reports. We followed the Strengthening the Reporting of Observational Studies in Epidemiology (STROBE) reporting guideline.

**Figure 1.**
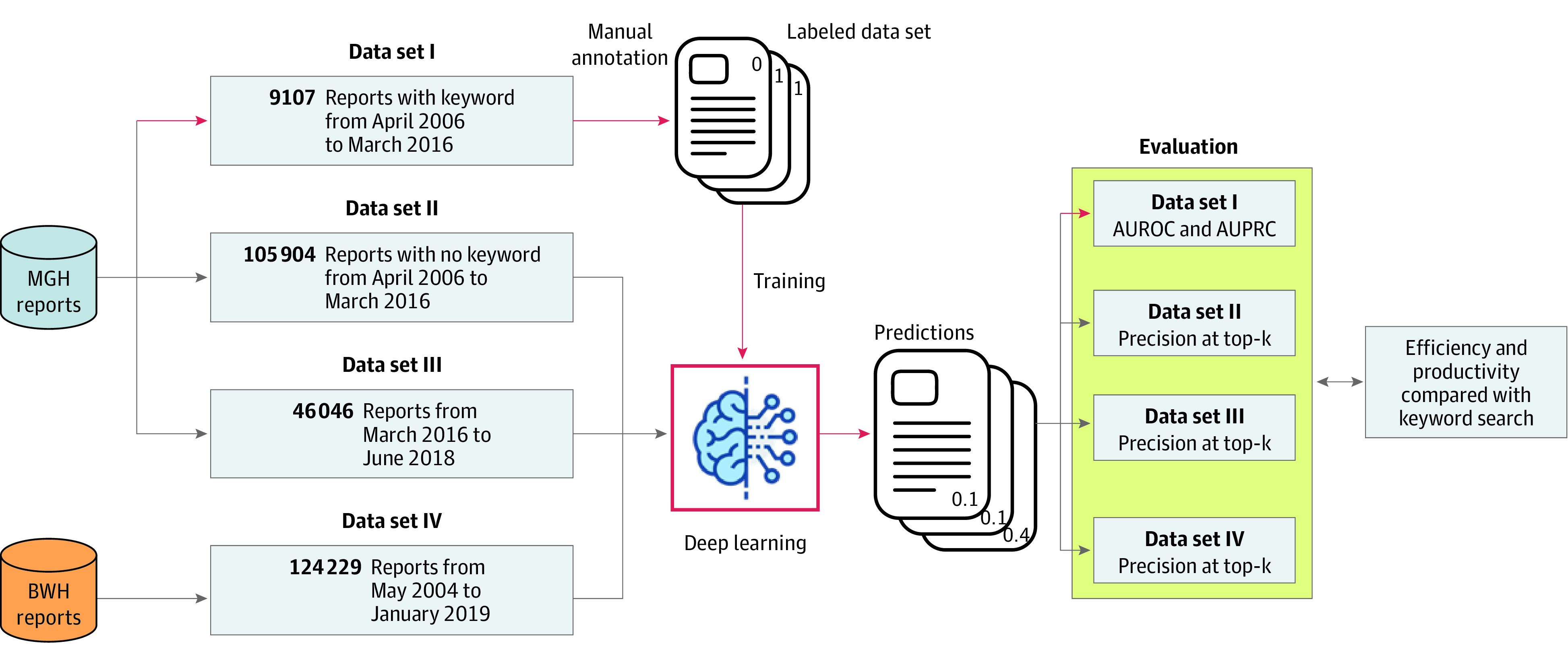
Study Data Sets and Overall Design This diagram depicts the 4 data sets used in this study, including the number of reports in each data set and when these reports were filed. Three data sets were from Massachusetts General Hospital (MGH), and 1 data set was from Brigham and Women’s Hospital (BWH). Data set I was used to train the deep learning model, whereas data sets II, III and IV were used to assess model performance and generalizability. AUPRC indicates area under the precision-recall curve; and AUROC, area under the receiver operating characteristic curve.

We collected hospital safety reports on patients from 2 academic medical centers: Massachusetts General Hospital and Brigham and Women’s Hospital. Massachusetts General Hospital reports were filed from April 2006 to June 2018, whereas Brigham and Women’s Hospital reports were filed from May 2004 to January 2019 (Figure 1). All staff at both institutions can file a safety report using software (RLDatix). Approximately 20 000 reports are filed annually at Massachusetts General Hospital and about 12 000 are filed annually at Brigham and Women’s Hospital. Although safety reports include several fields of coded data (eg, injury yes/no) that can be easily summarized and fed back to health care teams in a timely manner, many event details are entered in a free-text description field, which we used to identify allergic reactions in this study. Because safety report data are peer review–protected, the present study used the minimum patient and clinician details required with data that are appropriately labeled and securely handled and stored. Patient sex, race, and ethnicity were derived from the institutions’ electronic health record system.

### Deep Learning Algorithm Design

An event was considered to be an allergy event if a patient experienced 1 or more allergic reactions. Allergic reactions included those reactions with signs or symptoms that may have an immunologically mediated mechanism to a drug or may have a nondrug culprit (eg, food). Immunologically mediated mechanisms included all hypersensitivity reaction types^24^ and non–IgE-mediated or direct mast cell reactions that are clinically indistinguishable from allergic reactions.

We treated allergic reaction identification in safety reports as a document classification task and developed a 4-layer attention-based DNN to ascertain the likelihood of a report being an allergic reaction. We converted the free-text description of each report into a sequence of words before feeding it into the neural network (eFigure 1 in the Supplement). The first layer was a character-level encoder, which encoded the character sequence within each word using a single layer convolutional neural network (CNN)^25^ and a max-pooling function to create a fixed-dimension vector for the word. In the second layer, each word vector was concatenated with the word’s embedding,^26^ and a bidirectional long short-term memory (LSTM) network^27^ was built to use the contextual information of the entire report to generate an output vector for each word. Because different words within a report may have different levels of contribution in distinguishing the report, we added an attention model^28,29^ as a third layer to assign a unique weight to each word. The representation of a report was calculated using the weighted sum of all of the word representations within the report, where the weight of each word was the attention weight calculated in the third layer. The report representation was fed into the fourth layer, the classifier layer, which was trained using the cross-entropy loss function and the stochastic gradient descent optimizer.^30^ The output of the classifier was a vector representing the probability of a report being described as an allergy event. The attention-based DNN model was implemented using PyTorch, version 1.0 (PyTorch).^31^

### Training and Validation Data Sets

We constructed 4 data sets for the development and evaluation of the attention-based DNN model (Figure 1). Data sets I to III contained reports from Massachusetts General Hospital, whereas data set IV contained reports from Brigham and Women’s Hospital. Data set I, which was developed in a previous study,^12^ included 9107 reports retrieved by 101 expert-curated keywords (eTable 1 in the Supplement) from safety reports filed between April 2006 and March 2016. Two of us (N.A.P., C.M.M.) were trained to annotate the reports with an interannotator agreement (Cohen κ score) of 0.82.^4,32^ One of us (C.M.M.) labeled each report as an allergy event or not an allergy event, and a board-certified allergist or immunologist (K.G.B.) provided verification. We used this labeled data set to develop and validate the attention-based DNN model.

Data set II, containing 105 904 reports filed from April 2006 to March 2016 at Massachusetts General Hospital, excluded reports containing the 101 keywords and their morphological or lexical variations (eg, suffix [eg, -cillin], uppercase); we used this data set to assess the model’s ability to identify allergic reactions missed by the keyword-search approach. Data set III, including 46 046 reports filed between March 2016 and June 2018 at Massachusetts General Hospital, was used to assess the model’s generalizability to new cases from the same hospital. Data set IV, including 124 229 reports filed between May 2004 and January 2019 at Brigham and Women’s Hospital, was used to test the model’s reproducibility when applied to data from a different hospital.

### Model Evaluation

We assessed the attention-based DNN model’s performance on predicting allergic reactions using data set I with 5-fold cross-validation. We used the area under the receiver operating characteristic curve (AUROC) to assess and demonstrate the trade-off between sensitivity and specificity of the model across varying decision thresholds. We also generated the area under the precision-recall curve (AUPRC) to provide complementary information to the AUROC in the imbalanced classification.^33^

We evaluated the generalizability by applying the model to data sets II to IV. The model ranked the reports in descending order by their predicted probability of being an allergy event. We reported the model’s performance using precision at top-k, defined as the proportion of reports in the top-k set that were allergy events according to expert review. We generated a precision curve for the top-1000 model-identified reports for each data set. We further compared the deep learning approach (the model) with conventional keyword-search approach in terms of manual review effort (efficiency) and positive case yield (productivity). In this study, *positive* is defined as confirmed case of allergic reaction and *negative* as case of no allergic reaction. Details are described in the eMethods in the Supplement.

The attention-based DNN layer assigned each input word with a weight that measured the model’s attention when predicting allergic events. Attention value was an important means to interpret the model’s prediction, and we used it to extract words and phrases with high attention (eMethods in the Supplement). We also compared those model-identified high-attention words with the 101 expert-curated keywords to identify a list of new keywords extended by the model.

We investigated the severity of all of the validated allergic events in data sets II to IV; severity was coded according to 4 levels (no harm, minor harm, major harm, and death) by master’s or doctorate level–prepared nurses using a standardized scale modified from the Medical Expense Reimbursement Plan. We also reported the frequencies of common allergic reactions in the validated cases (eMethods in the Supplement).

### Statistical Analysis

Data analysis was performed between March 1, 2019, and July 18, 2020. Both AUROC and AUPRC were computed using the scikit-learn Python library (scikit-learn Developers).^34^ We estimated 95% CI using 2000 bootstrap iterations (Python, version 3.7; Python Software Foundation).

## Results

This study included 299 028 safety reports of 172 854 patients, with a median (range) of 1.6 (1-54) reports per patient. Of these patients, 86 544 were women (50.1%) and 80 319 were men (46.5%), with a median (interquartile range [IQR]) age of 59.7 (43.8-71.6) years. The free-text description contained a median (IQR) of 48 (25-84) words. Table 1 shows detailed patient and safety report characteristics by data set and hospital.

**Table 1.** Characteristics of the Hospital Safety Reports, Patient Population, and Data Sets for Machine Learning Model Development and Validation

Characteristic^a^	No. (%)					
	MGH				BWH	Total
	Data set I annotated (with keywords)	Data set II (without keywords)	Data set III (recent reports)	All MGH reports	Data set IV (all BWH reports)	All reports
Years	April 2006-March 2016	April 2006-March 2016	March 2016-June 2018	April 2006-June 2018	May 2004-January 2019	BWH: May 2004-January 2019 MGH: April 2006-June 2018
Patients^b^	7630	63 768	27 922	97 778	75 076	172 854
All reports^c^	9107	105 904^d^	46 046	174 799	124 229	299 028
Reports of identifiable patients^e^	9047	94 692	42 454	157 824	118 764	276 588
No. of reports per patient, mean (range)^f^	1.2 (1-12)	1.5 (1-54)	1.5 (1-34)	1.6 (1-54)	1.6 (1-40)	1.6 (1-54)
No. of words per reports, median (IQR)	74 (43-124)	51 (30-86)	63 (35-106)	57 (33-96)	37 (17-67)	48 (25-84)
Patient demographics						
Age, median (IQR), y^g^	58.3 (38.6-71.5)	59.3 (43.4-71.9)	60.1 (43.6-71.7)	59.3 (43.0-71.6)	60.2 (44.7-71.6)	59.7 (43.8-71.6)
Sex						
Female	3504 (45.9)	30 823 (48.3)	13 594 (48.7)	47 891 (49.0)	38 653 (51.5)	86 544 (50.1)
Male	3977 (52.1)	31 715 (49.7)	13 859 (49.6)	48 016 (49.1)	32 303 (43.0)	80 319 (46.5)
Unknown	149 (2.0)	1230 (1.9)	469 (1.7)	1871 (1.9)	4120 (5.5)	5991 (3.5)
Race						
White	5999 (78.6)	50 043 (78.5)	21 617 (77.4)	76 322 (78.1)	53 736 (71.6)	130 058 (75.2)
Black	415 (5.4)	3543 (5.6)	1742 (6.2)	5481 (5.6)	6832 (9.1)	12 313 (7.1)
Asian	228 (3.0)	1956 (3.1)	1048 (3.8)	3264 (3.3)	1877 (2.5)	5141 (3.0)
Others	94 (1.2)	841 (1.3)	280 (1.0)	1213 (1.2)	613 (0.8)	1826 (1.1)
Unknown	894 (11.7)	7385(11.6)	3235 (11.6)	11 498 (11.8)	12 018 (16.0)	23 516 (13.6)
Ethnicity						
Non-Hispanic	6605 (86.6)	55 408 (86.9)	24 079 (86.2)	84 579 (86.5)	62 271 (82.9)	146 850 (85.0)
Hispanic	588 (7.7)	4802 (7.5)	2298 (8.2)	7610 (7.8)	5417 (7.2)	13 027 (7.5)
Unknown	437 (5.7)	3558 (5.6)	1545 (5.5)	5589 (5.7)	7388 (9.8)	12 977 (7.5)

Abbreviations: BWH, Brigham and Women’s Hospital; IQR, interquartile range; MGH, Massachusetts General Hospital.

^a^ Summary of the characteristics of patient demographics information and cases.

^b^ Patients with a complete and valid medical record number.

^c^ Reports including those with and without a valid patient medical record number.

^d^ The sum of the 3 data sets from MGH is not equal to the total number of all reports because of the following reason. In a previous study in which data set I was created,^12^ exact keyword matching with a gradually curated keyword list was used to create the data set; thus, some cases, which contained morphological or lexical variations of the keywords, were missed. Therefore, in this study, to conduct a strict evaluation of the model’s ability to identify allergic reactions missed by keyword search, we constructed data set II using a more comprehensive keyword-matching algorithm. We excluded all the reports that contained any of the expert-curated keywords and morphological or lexical variations of the keywords (eg, prefix [eg, allerg-], suffix [eg, -cillin] and letter cases such as uppercase, lowercase, or capitals). Because of this reason, data set I plus data set II was less than all of the MGH reports between April 2006 and March 2016.

^e^ Reports linked to a valid patient medical record number.

^f^ Calculated using the reports linked to a valid patient medical record number.

^g^ Calculated using the event date and patient’s date of birth.

**Figure 2.**
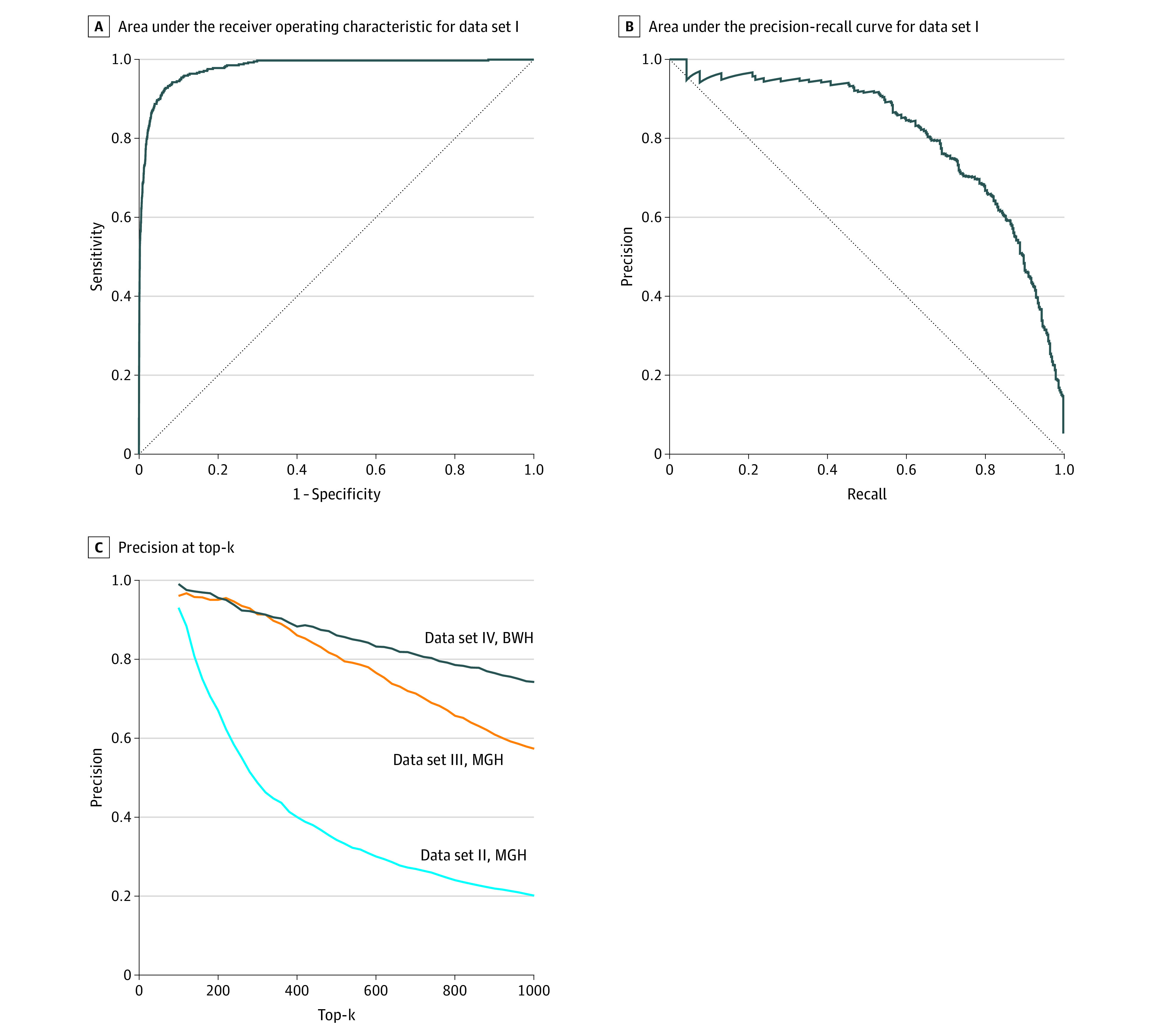
Deep Learning Model Performance BWH indicates Brigham and Women’s Hospital; and MGH, Massachusetts General Hospital.

The attention-based DNN model achieved an AUROC of 0.979 (95% CI, 0.973-0.985) and an AUPRC of 0.809 (95% CI, 0.773-0.845) in data set I (Figure 2A and B). The model achieved precisions of 0.930 at the top 100 and 0.201 at the top 1000 model-identified cases in data set II, precisions of 0.960 at the top 100 and 0.573 at the top 1000 in data set III, and precisions of 0.990 at the top 100 and 0.742 at the top 1000 in data set IV (Figure 2C).

From data sets II to IV, the model identified a total of 9411 possible cases that required manual review, of which 2378 (25.3%) were true allergic reactions (Table 2). The keyword-search approach extracted 26 027 cases (2.8 times as many cases as the model identified). Among the random 1000 cases from the keyword-search–only subset, no positive cases were found. In total, 1914 (7.4%) were estimated to be true.

**Table 2.** Model Efficiency and Productivity^a^

Data set	Measures	Keyword-search approach	Attention-based DNN model
II	Cases to review	0	1627
	True cases	0	184
	Precision, %	NA	11.3
III	Cases to review	10 131	1984
	True cases	570	625
	Precision, %	5.6	31.5
IV	Cases to review	15 896	5800
	True cases	1344	1569
	Precision, %	8.5	27.1
Total	Cases to review	26 027	9411
	True cases	1914	2378
	Precision, %	7.4	25.3

Abbreviations: DNN, deep neural network; NA, not applicable.

^a^ This table demonstrates the efficiency (ie, Cases to review—number of identified cases requiring manual review), productivity (ie, True cases—number of positive cases yielded), and precision (ie, positive predictive value; the proportion of true cases among all identified cases) of the attention-based DNN model compared with the keyword-search approach in data sets II, III, and IV (see eMethods in the Supplement for details).

Error analysis for both approaches is detailed in eTables 2 and 3 in the Supplement. The keyword-search approach failed largely because of lexical variations, incomplete keyword list, and contextual information (eg, negation, history of). The model failed for various reasons. For example, some cases contained insufficient information for being confirmed as allergic reactions. Nonallergic reactions can have similar symptoms (eg, rash caused by fungal or viral infections).

**Figure 3.**
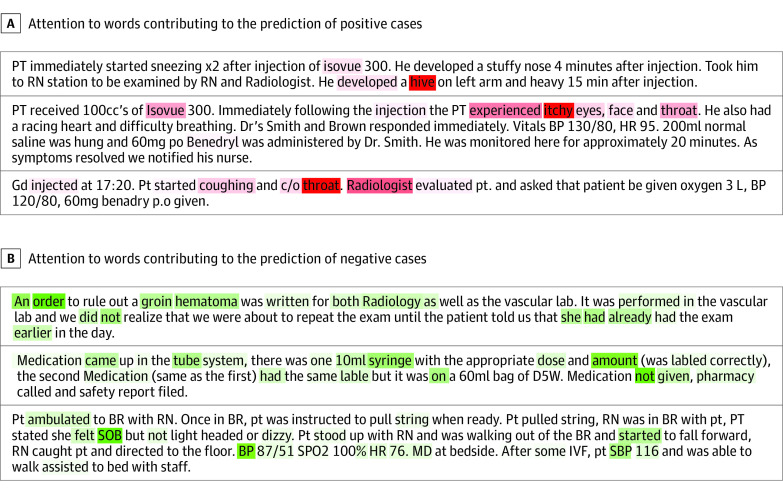
Attention Heat Maps These attention heat maps show how much attention the model gives to which words when making predictions of positive and negative cases of allergic reaction. Darker colors represent a higher attention weight. A, The words associated with prediction of positive cases included *itchy*, *hive*, and *throat*. The model captured misspellings (eg, *Benedryl* for *Benadryl*) and lexical variations (eg, *hive* for *hives*). B, The words associated with prediction of negative cases included *order*, *SOB (shortness of breath)*, *BP (blood pressure)*, and *not*. Details of individual cases were modified to preserve anonymity; no modifications affected the weights shown in this heat map.

Through the attention mechanism, we extracted 118 words with high attention weights, of which 19 (16.1%) overlapped with the expert-curated keywords and 99 (83.9%) were identified by the model only (eTable 1 in the Supplement). The overlapping keywords were common allergens (eg, *latex*) and reactions (eg, *rash*), whereas the model-identified additional keywords included diverse reactions (eg, *erythema*), allergens (eg, *Isovue*), misspellings (eg, *Benedryl*), and lexical variations (eg, *hive*). The heat maps in Figure 3 demonstrate how much attention the model gives and to which words when making predictions of positive and negative cases of allergic reaction. eFigure 2 in the Supplement illustrates the importance and frequency of allergic reaction keywords created by clinical experts and detected by the model. A set of more interpretable key phrases (eg, *throat tightness*) with large attention weights are shown in eTable 4 in the Supplement.

Among the 2378 validated allergic reaction events in data sets II to IV, 565 (23.8%) were associated with no harm to patients, 1798 (75.6%) with minor harm, 10 (0.5%) with major harm, and 2 (<0.1%) with death; 3 events had unknown severity. The most common allergic reactions were hives (859 [36.1%]), itching (483 [20.3%]), rash (371 [15.6%]), erythema or flushing (148 [6.2%]), angioedema (132 [5.6%]), and respiratory symptoms (48 [2.0%]) (eTable 5 in the Supplement).

## Discussion

This study demonstrated that a DNN that integrated CNN, LSTM, and an attention mechanism trained using a small set of keyword-identified, manually labeled safety reports can be accurate and useful in identifying allergic reactions from free-text descriptions in a large set of safety reports. The model performance, AUROC of 0.979 and AUPRC of 0.809 in data set I, showed its great capacity for detecting relevant signals from free-text narratives to make accurate predictions. The generalizability of the model was thoroughly evaluated using 3 data sets that were distinct from the original training data set. Given that allergic reaction is rare among all safety reports (approximately 1%-2%), the model demonstrated excellent ability (precision at top 100 > 0.90) in identifying allergic reactions from safety reports regardless of keywords, time frame, and institution.

Natural language processing (NLP) and machine learning have facilitated many health care–related tasks, such as cohort or case identification and outcome prediction.^35^ Although previous studies used NLP and machine learning in ADR detection,^36,37,38^ few have focused on detecting allergic reactions specifically. For example, one of the tracks in the 2018 National NLP Clinical Challenges shared tasks focused on identifying potential adverse drug events mentioned in clinical notes.^39^ Deep learning has also been used to detect ADRs using Twitter text.^23^ The 10% to 20% of ADRs that are allergic reactions often have specific clinical manifestations and causative culprits. Furthermore, their accurate diagnosis and documentation is critical to patient safety; allergic reactions predictably recur and may worsen with repeat exposure.^40^ To our knowledge, this study is the first investigation that successfully used deep learning to identify allergic reactions in safety reports.

The deep learning model was able to decrease the number of cases to review during the actual case detection from a large data set and overcame the low sensitivity associated with using the keyword-search approach. Compared with keyword search, the attention-based DNN model reduced the number of cases that required manual review from 26 027 to 9411 (63.8% lower) while identifying 464 more positive cases (1914 to 2378 [24.2% higher]), thus showing higher efficiency and productivity in identifying allergic reactions. In addition, the model took into consideration lexical variations within clinical documents (eg, synonyms, abbreviations, and misspellings) and incorporated a character CNN layer within the hierarchical neural network to handle this challenge. We also used LSTM to handle the contextual information surrounding words. This study demonstrated that this design can handle the language variations commonly used in free-text clinical details.

Although deep learning models are often regarded as a black box, by adding an attention layer, the model enables the predictions to be interpretable. For example, the heat maps in Figure 3 demonstrate that the model focused on words related to allergic symptoms (eg, *itchy*), body locations (eg, *throat*), and common allergic reaction culprit agents (eg, *Isovue*) when making predictions of positive cases. The model focused on information that was not relevant to allergy specifically (eg, orders, general vital signs, and negation terms) when making predictions of negative cases of allergic reaction. Although both model-identified keywords and expert-curated keywords included some of the most common and important words (eg, *Benadryl*, *rash*, and *hives*) that were predictive for allergy event identification (eFigure 2 in the Supplement), the model-extracted keyword list was more complete. Some expert-curated keywords, such as *anaphylaxis*, *urticaria*, and *angioedema*, were not highlighted by the model likely because of their low occurrence in the training set. Instead, these words’ high-occurrence synonyms (*anaphylactic*, *hives*, and *edema*) were successfully captured by deep learning. With the attention layer, the model was also able to extend the expert-curated keywords by detecting their misspellings and lexical variations as well as other important keywords that were not considered by the specialists. Because the character CNN layer can capture the similarity between character sequences, the model was able to extract some common misspelled keywords (eg, *Benedryl* vs *benadryl*). These novel features enhance model transparency while augmenting the clinical knowledge base.

Because the model was trained on the free-text descriptions written by a variety of health care team members, it might have similar potential for other free-text data sources such as clinical notes. Clinical narratives in safety reports are markedly similar to clinical narratives in all free-text health care documentation. Should the model perform similarly across data types, it may be able to be used for real-time allergy detection in hospital settings. After it is developed and trained, the model could detect true allergic reactions more efficiently than manual review, facilitating possible real-time applications to improve allergy documentation and clinical follow-up. Rapid detection of allergic reactions could allow the delivery of expeditious and appropriate care to patients with allergic reactions in health care settings. In addition, identical methods are likely extensible to other case identification tasks across a multitude of health care domains. This study demonstrates the promise of deep learning in improving patient safety efforts with the use of automated surveillance.

### Limitations

This study has several limitations, although the data used were obtained from 2 academic medical centers over a large study horizon (12 years for Massachusetts General Hospital and 15 years for Brigham and Women’s Hospital). First, the data were voluntarily reported; voluntary reporting captures just 1 in 10 ADRs.^41,42^ Although we do not know whether the events not reported were different in their free-text composition from the free-text descriptions in reported events, this model reassuringly performed well across time and setting, which limits the impact of reporting bias. Second, given that the model was trained on an imbalanced data set with a low rate of positive cases, the model was likely more prone to identifying negative cases. The AUROC and AUPRC measured for the present data sets may not be generalizable when applying the model to data sets with different ratios of positive or negative cases. Although we tried to oversample true positive cases during the model training stage, model performance did not improve. The AUROC and AUPRC on the test data sets were unclear because of the large amount of labeling efforts needed. Without a labeled test data set, we used precision at top-k as an alternative measure to evaluate model performance. Third, given the rarity of allergic reactions and the high cost of dedicated manual review, the model was trained on reports stratified by expert-curated keywords, which may be subject to human and sampling biases; despite this situation, the model achieved strong performance in identifying allergic reactions from reports that did not contain any expert-curated keywords. Still, the model’s focus on the original expert keywords might lead to some critical words or phrases being missed.

## Conclusions

This study demonstrated that a deep learning model that was trained on a small subset of safety reports can accurately and efficiently identify allergic reactions and can be generalized across the presence or absence of keywords, across time, and across hospitals. After validation on other forms of clinical data free-text description, such as clinical notes, this model could be applied to improve allergy care in health care settings and assessed in other patient safety domains, potentially enabling real-time event surveillance and guidance for medical errors and system improvement.^21,22^

## Notes

Supplement. **eMethods.** **eFigure 1.** Overall Framework of the Deep Learning Model **eFigure 2.** Allergy Keywords **eTable 1.** Expert-Curated Keywords Versus Deep Learning Detected Keywords **eTable 2.** Reasons and Examples Why Keyword Search Failed **eTable 3.** Example False Positive Cases in the Top-k Reviewed Cases that the Deep Learning Model Predicted With a Relative High Probability of Being an Allergy Event **eTable 4.** Key Phrases Automatically Detected by the Deep Learning Model **eTable 5.** Most Common Allergic Reactions (n = 2378)
