# Supplementary material for: Development and Validation of a Deep Learning Model for Detection of Allergic Reactions Using Safety Event Reports Across Hospitals
Source: JAMA Netw Open. 2020 Nov 16;3(11):e2022836. doi: 10.1001/jamanetworkopen.2020.22836 (PMC7670315; doi:10.1001/jamanetworkopen.2020.22836)
Supplement: Supplement. — eMethods. eFigure 1. Overall Framework of the Deep Learning Model eFigure 2. Allergy Keywords eTable 1. Expert-Curated Keywords Versus Deep Learning Detected Keywords eTable 2. Reasons and Examples Why Keyword Search Failed eTable 3. Example False Positive Cases in the Top-k Reviewed Cases that the Deep Learning Model Predicted With a Relative High Probability of Being an Allergy Event eTable 4. Key Phrases Automatically Detected by the Deep Learning Model eTable 5. Most Common Allergic Reactions (n = 2378) [file jamanetwopen-e2022836-s001.pdf]

Supplementary Online Content

Yang J, Wang L, Phadke NA, et al. Development and validation of a deep learning model for detection of allergic reactions using safety event reports across hospitals. *JAMA Netw Open*. 2020;3(11): e2022836. doi:10.1001/jamanetworkopen.2020.22836

eMethods.
eFigure 1. Overall Framework of the Deep Learning Model
eFigure 2. Allergy Keywords
eTable 1. Expert-Curated Keywords Versus Deep Learning Detected Keywords
eTable 2. Reasons and Examples Why Keyword Search Failed
eTable 3. Example False Positive Cases in the Top-k Reviewed Cases that the Deep Learning Model Predicted With a Relative High Probability of Being an Allergy Event
eTable 4. Key Phrases Automatically Detected by the Deep Learning Model
eTable 5. Most Common Allergic Reactions (n = 2378)

This supplementary material has been provided by the authors to give readers additional information about their work.

eMethods.

Efficiency and Productivity

We compared our deep learning approach to conventional keyword search in terms of manual review effort (efficiency) and positive case yield (productivity). To compare the efficiency, we determined the number of reports requiring manual review in each approach. For the ADNN model, we used a false negative rate of 0.5% as a cut-off to decide the number of reports, n, requiring manual validation; that is, we manually reviewed top-n reports identified from Datasets II-IV until the last 200 reviewed reports contained only one positive case. For the keyword-search approach, we used the 101 expert-curated keywords to identify all possible cases from Datasets II-IV and calculated the number of cases requiring manual review.

To compare the productivity, we determined the numbers of true cases identified by the two approaches. For the ADNN model, it was the number of cases among the top-*n* reviewed reports manually labeled as allergic reactions. For the keyword search, we estimated the number of true cases as follows: for each dataset of Dataset III and Dataset IV, we split all reports extracted by keyword-search into two subsets, the first containing reports that overlapped with those also identified by ADNN and the second containing reports identified only by keyword search. For the first subset, we used the number of the positive cases manually reviewed when evaluating the model. For the second subset, we estimated the number of positive cases based on the precision of the keyword-search approach on 1000 randomly selected reports, which were manually reviewed. That is, we first manually reviewed 1000 randomly selected cases identified by keywords and calculated the precision. We then estimated the number of positive cases ($\boldsymbol{n_{pos\,\epsilon\,N}}$) by multiplying the precision by the total of number of cases within each dataset, which is denoted as the following

$$\boldsymbol{n_{pos\,\epsilon\,N}} \approx \frac{\boldsymbol{n_{pos\,\in 1000}}}{\mathbf{1000}} \times \boldsymbol{N}$$

where $\boldsymbol{n_{pos\,\in 1000}}$ is the number of positive cases among the 1000 randomly selected cases and N is the total number of cases within each subset.

Lastly, we summed the numbers of positive cases from the two subsets as the number of positive cases extracted by keyword search. For Dataset II, because these reports did not contain any keywords, so the number of positive cases retrieved by keywords was 0.

In addition, we calculated the precision (i.e., the proportion of true positives among the identified cases) of each approach in identifying allergic reactions for each dataset. Finally, we conducted an error analysis for both approaches and investigated major causes of errors.

Interpretability and Keyword Extension

The ADNN attention layer assigned each input word with a weight that measures how much attention the model gives and to which words when predicting allergic reactions. To identify the words with high attention weight, we selected reports with a greater than 0.8 probability of being allergic reactions. We extracted the words with an attention weight at least two standard deviations above the average weights within that report and generated a list of “high attention keywords” detected by the model. We compared the “high attention keywords” with the 101 expert-curated keywords to identify a list of new keywords extended by the model. We similarly identified a set of key phrases. For each selected report, we extracted the consecutive words with attention weight at least one standard deviation above the average weights within the report and aggregated key phrases from those reports.

Extraction of Common Allergic Reactions

There were 2378 validated allergic events in total in dataset II, III, and IV. We categorized these reactions

into groups (Table 2). Each allergic reaction group includes one or more reaction keywords (Table 2, column “Included Keywords”). We calculated the frequency of each allergic reaction as following: we counted the number of allergic events that included any keyword(s) in the “included keywords” column as the high attention keyword (attention weight was at least two standard deviations above the average weights within the report,), and then divided the above number by all validated true allergic events (i.e. 2378). Then we ranked all the reactions and reported the top 10 most common allergic reactions.

©2020 Yang J et al. *JAMA Network Open*

eFigure 1. Overall Framework of the Deep Learning Model

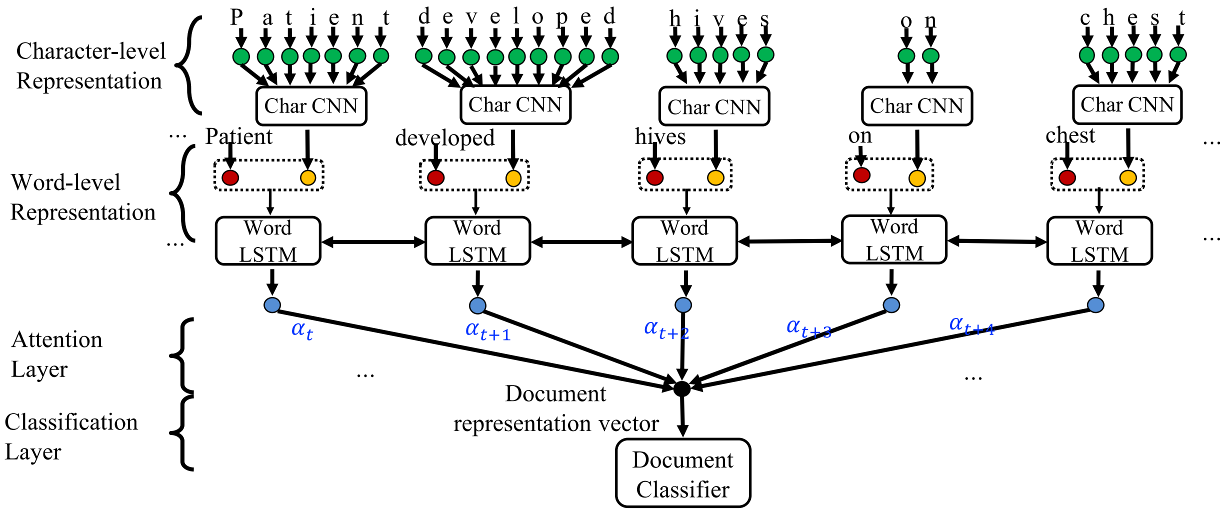

Green, yellow and red circles represent the character-level embeddings, character-sequence representation, and word-level embeddings, respectively. $\boldsymbol{\alpha_t}$ is the attention weight of the t-th word. The character-level representation encodes the character sequence of each word with a one-layer CNN. The output of character CNN is concatenated with the word embedding to build the word representation. The word representation is fed into a bidirectional LSTM to capture the context information of the sentence. An attention layer is applied on top of the word representation layer to calculate the attention weight for each word. The final report-level representation is the weighted sum of all the word vectors within the report, which considers the context of the report. The classifier was trained using the cross-entropy loss function and the Stochastic Gradient Descent (SGD) optimizer. The output of the classifier is a vector representing the probability of whether or not a report contained allergic reactions.

eFigure 2. Allergy Keywords

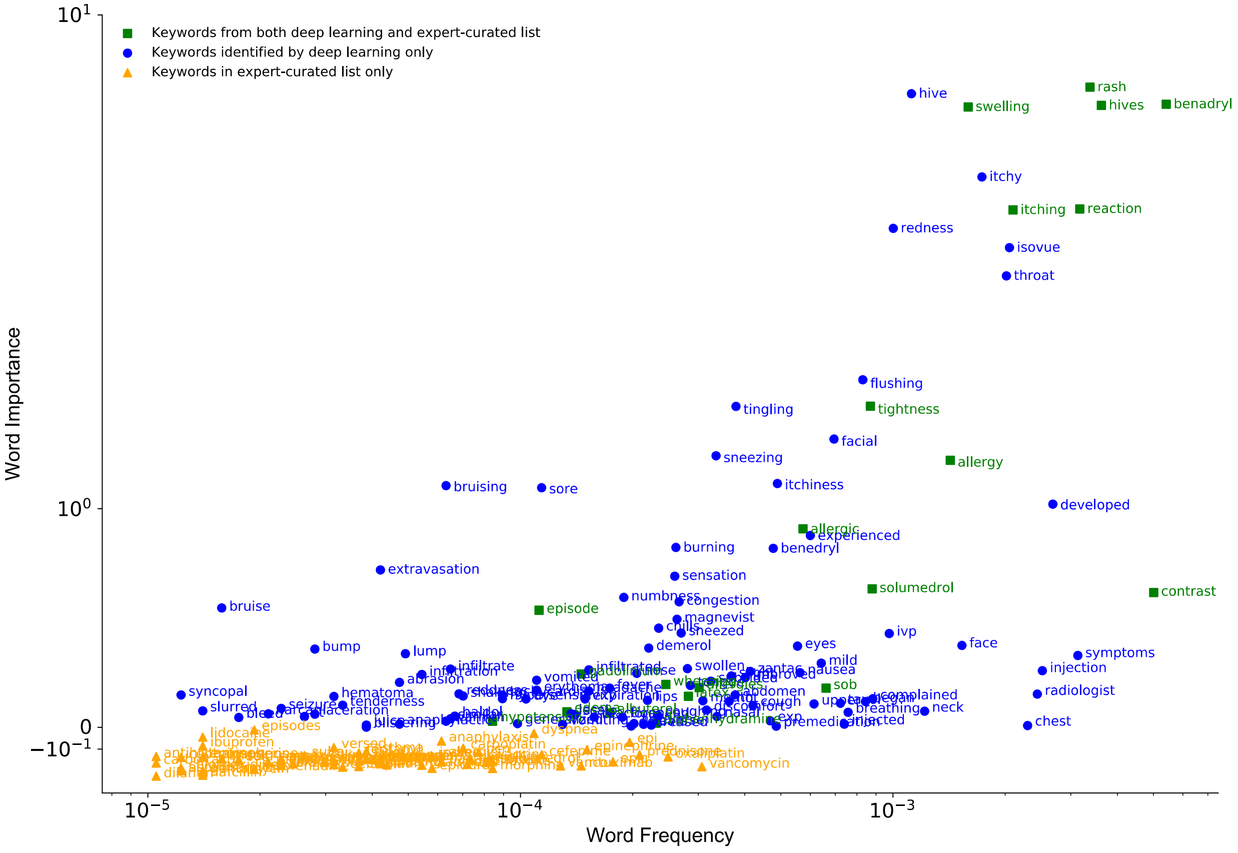

This graph illustrates the importance and frequency of allergic reaction keywords created by clinical experts and detected by the model. The word frequency was calculated by dividing the number of occurrences of a keyword by the total number of words in all reports with greater than 0.8 predicted probability of being an event of allergic reaction. The word importance is the average of a keyword’s attention weights in the reports with greater than 0.8 predicted probability in which it appeared. Green squares represent the overlapping keywords identified by both experts and the model. Yellow triangles represent keywords that were only included in the expert-curated list. Blue circles represent extended keywords only identified by the model’s attention mechanism. Additional details about these keywords are listed in eTable 1.

©2020 Yang J et al. *JAMA Network Open*

eTable 1. Expert-Curated Keywords Versus Deep Learning Detected Keywords[a]

	Keywords by Experts Only (82)	Overlapping Keywords (19)	Keywords by Deep Learning Only (99)
**Body Location**			throat, lip, lips, mouth, nose, eyes, nasal, face, facial, forehead, ears, scalp, neck, abdomen, chest, tongue
**Symptoms and Reactions**	rxn, anaphylaxis, angioedema, rhinitis, bronchospasm, asthma, dyspnea, shortness of breath, urticaria, pruritus, pruritis, closing, shock	allerg- (i.e., allergy, allergic, allergies), rash, hives, swelling, reaction, itching, tightness, sob, wheezing, episode, hypotension, edema	hive, burning, itchy, itchiness, sore, chills, sneezed, sneezing, symptoms, reddness, cough, coughing, breathing, reactions, fever, tenderness, nausea, vomiting, vomited, headache, flushing, tingling, sensation, congestion, bruise, bump, lump, swollen, infiltrate, infiltrated, infiltration, abrasion, laceration, erythema, tachycardia, syncopal, hypersensitivity, discomfort, anaphylactic, blistering, bruising, bleed, warmth, hot, numbness, shaking, hematoma, seizure, slurred, redness
**Allergy Culprit Agents**	vaccine, -caine, midazolam, versed, chlorhexidine, curium, fentanyl, morphine, dilaudid, heparin, lovenox, enoxaparin, dalteparin, fragmin, vancomycin, vanc-, penicillin, pcn, cef-, ceph-, -mycin, floxacin, -cillin, sulfa, sulpha, bactrim, tegretol, dilantin, carbamazepine, ritux, rituximab, carbo-, oxali-, -platin, taxol, aspirin, ibuprofen, motrin, advil, naproxen, naprosyn, iodine, phenytoin, food, milk, egg, fish, shellfish, crab, lobster, shrimp, nut, almond, cashew, pine, pecan, pistachio, wheat, soy	contrast, latex, gadolinium	isovue, dye, magnevist, ivp, radiologist, demerol, narcan, zantac, taxol, haldol, injection, exp, st, premedication, juice
**Anti-Allergy Treatment**	antihistamine-, anti-histamine, fexofen-, allegra, steroid, prednisone, prednisolone, pred, epi-, epi-pen	benadryl, diphen- (diphenhydramine), solu- (solumedrol), albuterol 4	benedryl
**Other**			experienced, began, less, mild, decreased, adverse, developed, extravasation, expiration, improved, similar, complained, upper, injected,

©2020 Yang J et al. *JAMA Network Open*

			slight, subsided, generalized

[a] "-xxx" represents the matching using "xxx" as the suffix, "xxx-" represents the matching using "xxx" as the prefix.

©2020 Yang J et al. *JAMA Network Open*

eTable 2. Reasons and Examples Why Keyword Search Failed[a]

Reasons for Keyword Search Failure	Examples[b]	Rate (%)
Symptom not specific to allergic reaction	Pt back on R side noted to have a reddened weepy area about 4 inch x 7 inch rectangular sized area. Pt c/o *itching/burning feeling* on site. PA assessed skin. *Suspect that it is a burn mark from heating pad used in OR.*	68
Adverse reaction, but not enough information to determine if allergic reaction	PT here for chemotherapy. PT received Decadron IV, Benadryl IV and Tylenol po prior to infusion. 30 min post SOI, pt developed rigors, denies SOB, chest pain, nausea or dizziness. Infusion stopped, MD notified. Infusion resumed at 8:30 at 10% of rate. At 9:08pm pt c/o thachcardia/hard breathing, denies SOB. Infusion stopped, MD notified. Given Solu-Medrol and Tylenol PO. Infusion resumed at 9:35.	8
History of allergic reaction (Stevens-Johnson syndrome – SJS)	Pt had an appointment to see a cornea specialist to *follow up her corneal involvement from SJS*, which has been an active issue while in the hospital.	7
Keyword term misspelled; Brand name of drug used; Symptom not included in keyword list	Stopped infusion 4 minutes into start of *Taxol*. Pt initially felt hot with flushing, then developed *lower back pain* and *dsypnea* (dyspnea).	5
Common allergen mentioned, but no evidence of allergic reaction	The patient arrived in clinic after a CT abdomen with IV contrast with his peripheral IV in place. He said he asked to have it left in, in case he needed to be admitted to the ED. I removed the line in clinic.	4
Negative terms not filtered by keyword search	Patient had bovie grounding pads on bilateral thighs. They were on the anterior-lateral portion of the thighs. Upon removal of the pads a reddened and purplish area was noted on the periphery where the grounding pads were. Area was *not* raised and patient *wasn't complaining* of any *itching* or pain.	3
Anti-allergy medications used for non-allergic reasons	Pt was on a *steroid* taper of IV *Solumedrol* to be changed to *prednisone* po. Pt. recieved IV dose of *Solumderol* at 9AM and should have recieved po *prednisone* at 9pm. The order was approved but the next dose was scheduled for 9pm on 9/26/10 instead of the appropriate time of 9pm on 9/25/10. The patient then missed thier evening dose of *prednisone*.	3
Not an adverse event, but contains allergic reaction terms	Patient stated no allergies to CT contrast dye and has had it in the past with no problems. No allergy was documented in the chart. Patient was given 17ml of iodinated contrast at 3:33pm and vomited immediately after. Patient drank water and said she felt ok and to continue with the exam. Radiologist believes this was a physiological reaction but wants the patient to wait 30 minutes after the exam to make sure he does not have a reaction. …	1
Allergic reaction plan for future	... Instructions in order said "Admin instructions: Vital signs per site Antibiotic Test Dose Policy. For intravenous test doses, RN to administer by IV push over 5 minutes. If no reaction to test dose, give full dose 30-60 min after per site policy. If rash or other symptoms develop, stop medication and treat with diphenhydramine 50 mg IV and notify RC. For anaphylactic reaction, follow site protocol. "	1

©2020 Yang J et al. *JAMA Network Open*

[a]100 failure cases were randomly selected and manually reviewed
[b]Reports modified slightly to anonymize patient, provider, and institution. Pt: patient. OR: operation room. IV: intravenous injection.

©2020 Yang J et al. *JAMA Network Open*

eTable 3. Example False Positive Cases in the Top-k Reviewed Cases that the Deep Learning Model Predicted With a Relative High Probability of Being an Allergy Event[a]

Reasons for Deep Learning Failure	Examples[b]	Rate (%)
Not an allergic reaction, but the report contains symptoms (e.g., rash), likely due to other clinical conditions[c]	Patient demonstrated a *rash* and dystonic movements which the team attributes to an increase in the dose of the Neurontin.	41
Adverse reaction that could be allergic, but there was no enough information for the reviewer to confirm that it was an allergic reaction (e.g., caused by a drug)	Patient developed *cutaneous rash*(only *redness*) over upper chest and right side of face and left arm.	25
Anti-allergy medications given for non-allergic indication	Pt was ordered for immunoglobulin yesterday, 30g approved by blood bank. Pt was premedicated with Hydrocort 100mg IV, Benadryl 50mg IV and Tylenol 650mg IV 30 min prior to transfusion.	16
Reference to prior allergy but no current allergic reaction	Patient allergic to kiwi, allergy listed in EHR (hives, facial swelling-updated after event to add anaphylaxis per pt); pt served lunch tray with kiwi on it. Pt requested tray be removed, in case of cross-contamination. Tray removed, new tray ordered.	10
Report mentions body part (e.g., throat) commonly involved in allergic reaction, but not allergic reaction	During the closing count, discovered missing half of the throat pack. 1 whole throat pack opened during the case and cut into half. the other half is missing. MD notified and denied inserting a throat pack to the patient. x-ray without evidence of throat pack on the patient.	5
Adverse reaction, but not an allergic reaction	Pt underwent a permanent pacemaker insertion; subsequently *developed a rash* to left axilla area; *area red* with yellow pustules; patient complaints of intermitten pain to affected area; physician assistant, physician notified.	2
Not an adverse event, but the report contained discharge instructions that listed some allergic reactions that the patient should watch for	Pt extremely impatient to leave hospital once he learned that he was medically stable to be discharged. Pt unwilling to wait additional minute to sign discharge instructions. Pt agreed to return to hospital if he experiences fever, increase in diarrhea, or other new symptoms such as *shortness of breath*, fever, *rash*, and other symptoms.	1

[a]100 failure cases were randomly selected and manually reviewed
[b]Reports modified slightly to anonymize patient, provider, and institution. bx: biopsy. PCA: patient care assistant. Pt: patient. Sxs: symptoms. VSS: vital sign stable.
[c]The patient likely had shingles, a viral infection that causes a painful rash, and was treated with Neurontin.

©2020 Yang J et al. *JAMA Network Open*

eTable 4. Key Phrases Automatically Detected by the Deep Learning Model

Groups	Phrases
**Symptoms and Reactions**	allergic reaction, itchy throat, facial flushing, sore throat, itchy rash, throat tightness, throat swelling, burning sensation, developed flushing, burning eyes, redness swelling, experienced sneezing, developed rigors, developed redness, developed numbness, developed fever, tingling sensation, tingling lips, throat symptoms, reaction itching, reaction hives, rash itching, nasal burning, mild tightness, mild burning sensation, itchy hive, feeling hot, facial redness, facial numbness, facial bruising, face flushing, face burning, eyes burning, experienced tingling, experienced slight, experienced congestion, experienced chest tightness, experienced burning, developed tightness, developed tachycardia, developed swollen, developed swelling, developed sob, developed reddness, developed nausea, developed itching, developed infiltration, developed facial flushing, developed facial, developed burning sensation, developed allergic symptoms
**Symptom improved**	symptoms subsided, slight improvement, nausea improved
**Contrast Media**	contrast isovue, contrast injection, gadolinium contrast

©2020 Yang J et al. *JAMA Network Open*

eTable 5. Most Common Allergic Reactions (n = 2378)

Reactions	Included Keywords[a]	n (%)
**Hives**	hives, hive, urticaria	859 (36.1)
**Itching**	itching, itchy, itchiness, pruritus, pruritis	483 (20.3)
**Rash**	rash	371 (15.6)
**Erythema or flushing**	erythema, flushing, warmth, hot, reddness, redness, burning	148 (6.2)
**Angioedema**	angioedema, swelling, edema, swollen	132 (5.6)
**Respiratory symptoms**	bronchospasm, tightness, wheezing, tenderness	48 (2.0)
**Sneezing**	sneezed, sneezing	35 (1.5)
**Gastrointestinal symptoms**	nausea, vomiting, vomited	26 (1.1)

[a] Allergic reactions were grouped based on the reaction keywords mentioned in the reports. These keywords included expert-curated keywords as well as those identified by the deep learning model.

©2020 Yang J et al. *JAMA Network Open*
